# MI-181 Modulates Cilia Length and Restores Cilia Length in Cells with Defective Shortened Cilia

**DOI:** 10.1021/acschembio.4c00186

**Published:** 2024-08-06

**Authors:** Ankur A. Gholkar, Thomas V. Gimeno, Jalie E. Edgemon, Myung Shin Sim, Jorge Z. Torres

**Affiliations:** † Department of Chemistry and Biochemistry, University of California, Los Angeles, California 90095, United States; ‡ Department of Medicine Statistics Core, University of California, Los Angeles, California 90095, United States; § Department of Medicine’s Division of General Internal Medicine and Health Services Research, University of California, Los Angeles, California 90095, United States; ∥ Jonsson Comprehensive Cancer Center, University of California, Los Angeles, California 90095, United States; ⊥ Molecular Biology Institute, University of California, Los Angeles, California 90095, United States

## Abstract

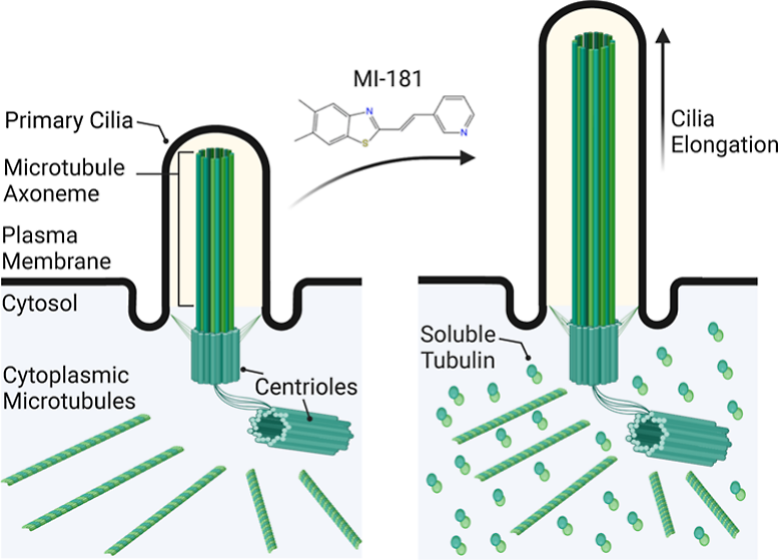

Primary cilia are membrane-covered microtubule-based structures that protrude from the cell surface and are critical for cell signaling and homeostasis during human development and adulthood. Dysregulation of cilia formation, length, and function can lead to a spectrum of human diseases and syndromes known as ciliopathies. Although some genetic and chemical screens have been performed to define important factors that modulate cilia biogenesis and length control, there are currently no clinical treatments that restore cilia length in patients. We report that the microtubule-targeting agent MI-181(mitotic inhibitor-181) is a potent modulator of cilia length and biogenesis. Treatment of retinal pigment epithelial-1 cells with MI-181 induced an increase in the average size of cilia and in the percent ciliated cells under nonstarved conditions. Importantly, MI-181 was effective at rescuing cilia length and ciliation defects in cells that had been treated with the intraflagellar transport inhibitor Ciliobrevin D or the O-GlcNAc transferase inhibitor OSMI-1. Most importantly, MI-181 induced an increase in cilia length and restored ciliation in cells with compromised shortened cilia at low nanomolar concentrations and did not show an inhibitory response at high concentrations. Therefore, MI-181 represents a lead molecule for developing drugs targeting ciliopathies characterized by shortened cilia.

Primary cilia are microtubule-based structures that protrude from the cell surface and are important for external sensing, signaling, and homeostasis of most postmitotic cell types.^1^ The ciliary axoneme, comprised of microtubules and associated proteins, is built from the mother centriole within the microtubule organizing center.^1^ Cilia formation requires the availability of soluble tubulin monomers and the intraflagellar transport (IFT) machinery, which functions as a microtubule-based transport system within cilia and is required for building, maintaining, and regulating the function of cilia.^2^ Mutation or dysregulation of the IFT system and other key structural and regulatory components of cilia formation, length control,^1^ and function leads to a group of diseases and syndromes collectively known as ciliopathies.^1^ Although much work has been done to understand the components, organization, and function of cilia, less work has been done to develop pharmacological interventions to treat ciliopathies.

Previous studies to understand ciliogenesis and cilia homeostasis have mainly focused on the genetic and molecular characterization of novel genes critical for these processes.^3−5^ For example, Failler et al.^4^ performed a genome-wide RNAi screen to define factors that negatively regulate ciliogenesis. Although fewer studies have been devoted to defining small molecules that can modulate cilia growth and function,^6,7^ several small-scale chemical genetic screens have been conducted to define small molecules that affect cilia size.^8−10^ For example, Khan et al.^10^ screened through 1600 compounds, at 10 μM concentration, to identify compounds that arrested CFPAC-1 cancer cells and induced ciliogenesis. However, hits from the study were not validated to ensure that they were targeting their intended targets and they did not assess whether hit compounds could restore cilia length in cells with shortened cilia. Therefore, there is a critical need to define novel small molecules that can restore cilia length and function as a means to develop novel therapeutics for the treatment of ciliopathies.

Interestingly, Sharma et al.^11^ showed that the levels of soluble cytosolic tubulin likely modulated the size of cilia. Treatment of cells with moderate levels of the microtubule depolymerizing drug nocodazole induced ciliogenesis and increased cilia length, while treatment with the microtubule stabilizer taxol inhibited ciliogenesis.^11^ However, nocodazole is primarily used as a research tool due to its toxic chemical properties, thus there is currently a need to define novel compounds that could be developed into ciliopathy therapeutics. Previously, we identified the small molecule MI-181 (5,6-dimethyl-2-[(*E*)-2-(pyridin-3-yl)ethenyl]-1,3-benzothiazole, aka mitotic inhibitor-181) as a novel potent inhibitor of cancer cell proliferation We further defined MI-181 as a microtubule-targeting agent that depolymerized cytoplasmic microtubules and mitotic spindle microtubules and arrested cells early during cell division.^12^ We then solved the protein-ligand cocrystal structure of MI-181 bound to β-tubulin, which showed that MI-181 bound near the colchicine binding site and had a unique binding mode and mechanism of action.^13^

Here, we have analyzed microtubule-targeting drugs that destabilize microtubules (MI-181, colchicine, nocodazole) or stabilize microtubules (taxol) for their effect on ciliogenesis and cilia length. We report that MI-181 is a modulator of ciliogenesis and cilia length. Treatment of human hTERT-RPE-1 cells with MI-181 led to an increase in the percent of ciliated cells and an increase in the length of cilia. Further, MI-181 rescued ciliogenesis and ciliary length defects caused by chemical inhibition of the IFT system or regulatory factors that are important for promoting ciliogenesis and establishing cilia length control. Therefore, MI-181 represents a promising molecule for the development of therapeutics to treat ciliopathies where ciliogenesis and cilia length regulation are perturbed.

## Results and Discussion

### MI-181 Modulates Cilia Length

**Figure 1:**
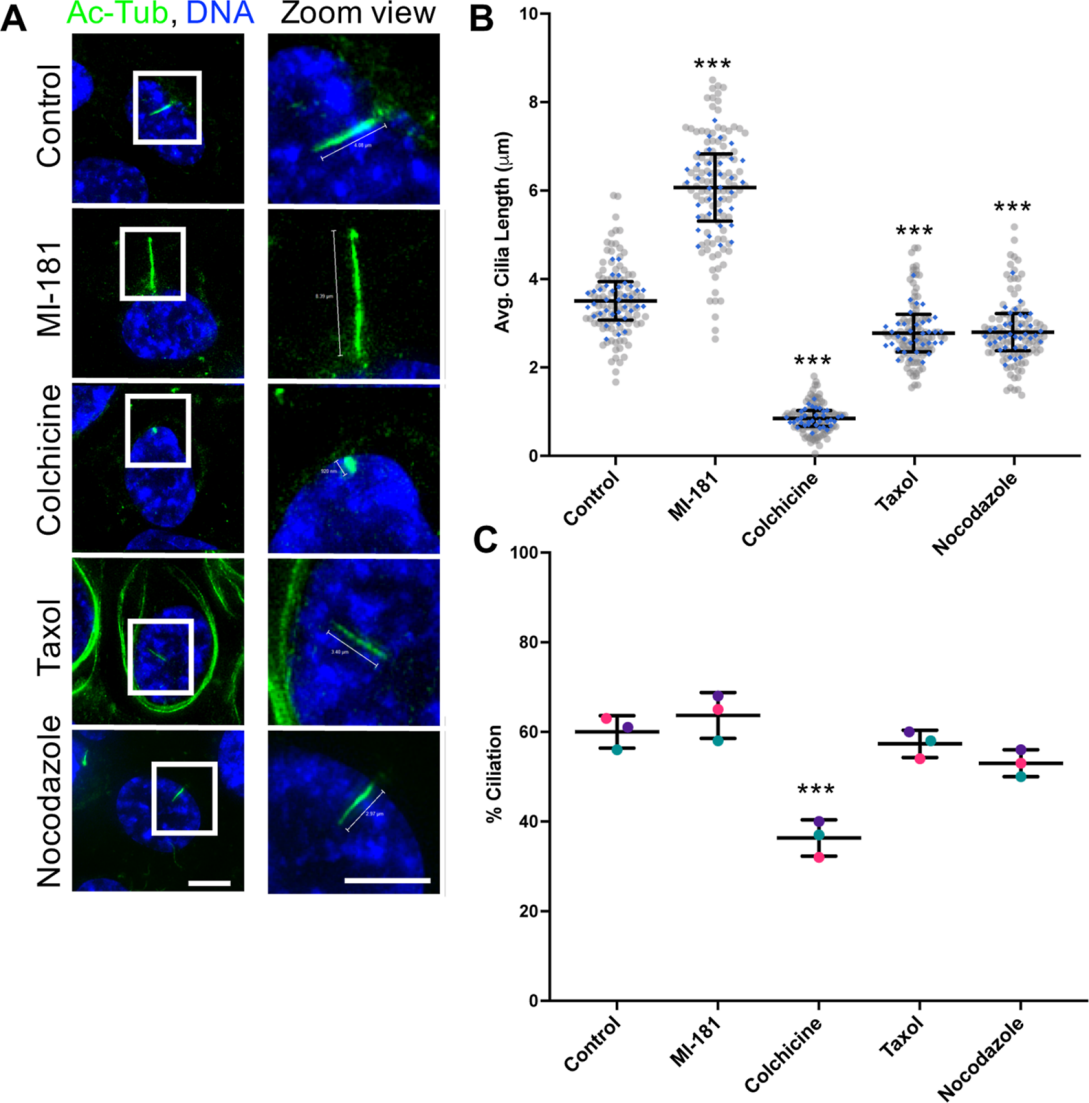
MI-181 modulates cilia length. (A) Immunofluorescence (IF) microscopy of hTERT RPE-1 cells treated with control vehicle DMSO, 100 nM MI-181, 10 μM colchicine, 100 nM taxol, or 116 nM nocodazole for 24 h after serum withdrawal, fixed, and costained for the cilia axoneme marker acetylated tubulin (antiacetylated tubulin antibody, green) and DNA (Hoechst 33342, blue). Right side panels show zoom view of the areas in the white boxes in the left panels. Scale bars indicate 5 μm. (B) Graph shows summary of the average length of cilia (*y*-axis) for each treatment (*x*-axis). (C) Graph shows summary of the percentage of ciliated cells (*y*-axis) for each treatment (*x*-axis). (B–C) Data is represented as the average ± SD. Asterisks indicate statistical significance as **p < 0.01 and ***p < 0.001 compared to control. See Quantification and Statistical Analyses section for details.

Inspired by the need to develop novel therapeutics for the treatment of ciliopathies, we sought to test the hypothesis that the microtubule-targeting agent MI-181 could be used to induce cilia formation and to restore cilia length in cells with defective shortened cilia. To begin to test this, we utilized human retinal pigment epithelial (hTERT RPE-1) cells, which undergo ciliogenesis upon starvation (serum withdrawal), and analyzed the effects of microtubule-targeting agents on cilia formation and cilia length. hTERT RPE-1 cells were treated with vehicle control DMSO, 100 nM MI-181, 10 μM colchicine, 100 nM taxol, or 116 nM nocodazole for 24 h after serum withdrawal. Cells were then fixed, costained for DNA, using Hoechst 33342, and the cilia axoneme marker acetylated tubulin (Ac-Tub), using an antiacetylated tubulin antibody, and analyzed by immunofluorescence (IF) microscopy. At these concentrations, nocodazole, colchicine, and taxol treatments led to a decrease in ciliated cells and a decrease in cilia length, while treatment with MI-181 led to a significant increase in the average length of cilia (DMSO = 3.52 ± 0.43 μm, MI-181 = 6.09 ± 0.76 μm, *p* < 0.001) and a slight, but not significant, increase in the percentage of ciliated cells (DMSO = 60 ± 3.61, MI-181 = 64 ± 5.13, *p* = 0.2) (Figure 1A–C). Interestingly, the treatment of nonstarved cells with MI-181 for 24 h also led to a significant increase in the average length of cilia (DMSO = 2.97 ± 0.43 μm, MI-181 = 4.75 ± 0.42 μm, *p* < 0.001) and a significant, increase in the percentage of ciliated cells (DMSO = 28.67 ± 3.21, MI-181 = 38.33 ± 2.52, *p* < 0.001) (Figure S1A–C). These results indicated that MI-181 promoted an increase in cilia length in both starved and nonstarved cells.

### MI-181 Modulates Ciliation and Cilia Length in a Concentration-dependent Manner

**Figure 2:**
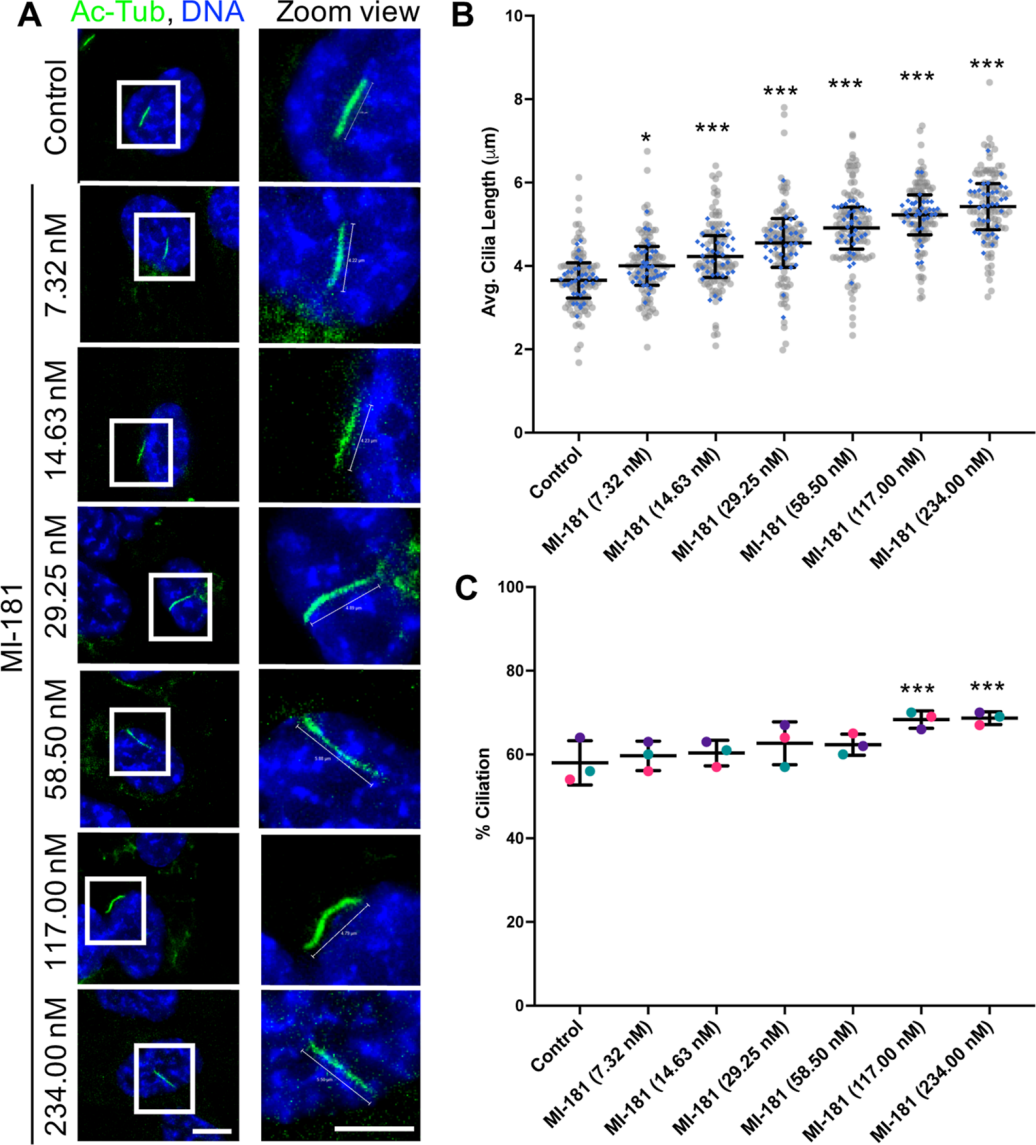
MI-181 modulates cilia length in a concentration-dependent manner. (A) IF microscopy of hTERT RPE-1 cells treated with control DMSO or increasing concentrations of MI-181 (7.32 nM to 234 nM) for 24 h after serum withdrawal, fixed, and costained for cilia (acetylated tubulin, green) and DNA (Hoechst 33342, blue). Scale bars indicate 5 μm. (B) Graph shows summary of the average length of cilia (*y*-axis) for each treatment (*x*-axis). (C) Graph shows summary of the percentage of ciliated cells (*y*-axis) for each treatment (*x*-axis). (B–C) Data is represented as the average ± SD. Asterisks indicate statistical significance as *p < 0.05 and ***p < 0.001 compared to control. See Quantification and Statistical Analyses section for details.

Previous results from Sharma et al.^11^ showed that nocodazole had a concentration-dependent effect on cilia formation and size, with low levels of nocodazole (1 nM and 10 nM) having no effect, while moderate levels (100 nM) increased ciliogenesis and cilia length, and high levels (1 μM) had a negative effect on ciliogenesis with no cilia formation. Therefore, we sought to determine if MI-181’s ability to increase cilia length was concentration-dependent. hTERT RPE-1 cells were treated with DMSO or increasing concentrations of MI-181 (7.32 nM to 234 nM) for 24 h after serum withdrawal and cilia were imaged by IF microscopy. A trend was apparent where increasing the concentration of MI-181 led to an increase in the average length of cilia (DMSO = 3.66 ± 0.43 μm; 7.32 nM MI-181 = 4.01 ± 0.47 μm, *p* < 0.05; 14.63 nM MI-181 = 4.24 ± 0.50 μm, *p* < 0.001; 29.25 nM MI-181 = 4.56 ± 0.59 μm, *p* < 0.001; 58.5 nM MI-181 = 4.92 ± 0.51 μm, *p* < 0.001; 117 nM MI-181 = 5.24 ± 0.48 μm, *p* < 0.001; 234 nM MI-181 = 5.44 ± 0.56 μm, *p* < 0.001) (Figure 2A, B). While there was only a significant change in the percentage of ciliated cells at higher concentrations of MI-181 (DMSO = 58 ± 5.29; 117 nM MI-181 = 68.33 ± 2.08, *p* < 0.001; 234 nM MI-181 = 68.67 ± 1.53, *p* < 0.001) (Figure 2C). A similar trend was observed when MI-181 was added for 2 h to cells that had already been starved for 24 h, with the exception that no increase in percent ciliation was observed during this short time period (Figure S2A–C). Interestingly, the addition of MI-181 to nonstarved cells for 24 h not only led to an increase in the average length of cilia but also the percentage of ciliated cells (Figure S3A–C). These results indicated that MI-181 induced ciliogenesis in nonstarved cells and promoted an increase in cilia length at low nM concentrations (14.63 nM) and in a concentration-dependent manner with no significant negative effect on cilia length even at a concentration of 234 nM, which was ∼10-fold higher than its previously reported IC_50_ for inducing cell death in cancer cells.^12^

### MI-181 Restores Ciliation and Cilia Length in Cells with Compromised Shortened Cilia

**Figure 3:**
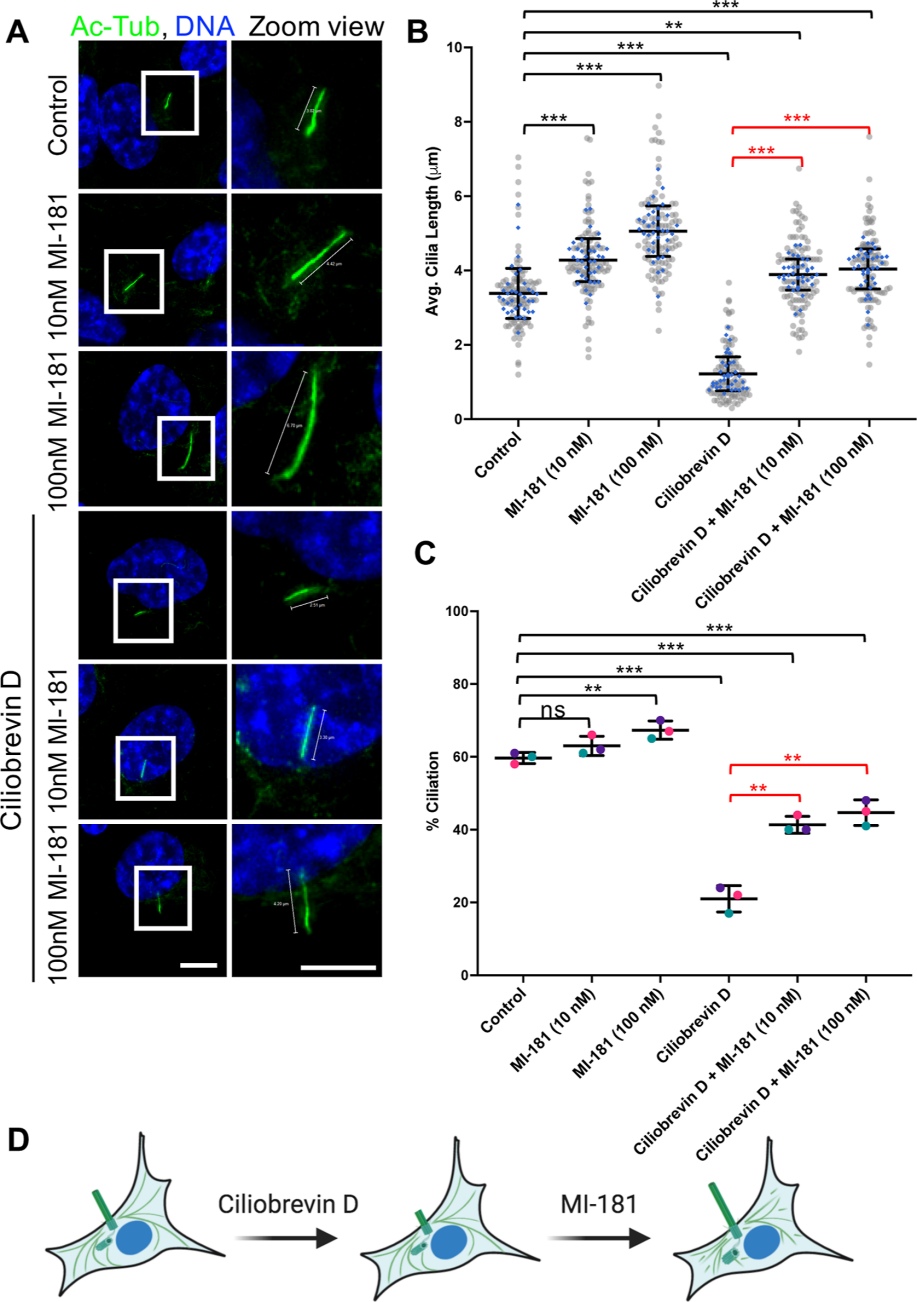
MI-181 restores ciliation and cilia length in cells with a compromised IFT system. (A) IF microscopy of hTERT RPE-1 cells treated with DMSO, 10 nM MI-181, 100 nM MI-181, 50 μM Ciliobrevin D, 50 μM Ciliobrevin D + 10 nM MI-181, or 50 μM Ciliobrevin D + 100 nM MI-181 for 24 h after serum withdrawal, fixed, and costained for cilia (acetylated tubulin, green) and DNA (Hoechst 33342, blue). Right side panels show zoom view of the areas in the white boxes in the left panels. Scale bars indicate 5 μm. (B) Graph shows summary of the average length of cilia (*y*-axis) for each treatment (*x*-axis). (C) Graph shows summary of the percentage of ciliated cells (*y*-axis) for each treatment (*x*-axis). (B–C) Data is represented as the average ± SD. Asterisks indicate statistical significance as **p < 0.01 and ***p < 0.001 compared to control. Not statistically significant is indicated by ns. See Quantification and Statistical Analyses section for details. (D) Schematic summary of the results.

**Figure 4:**
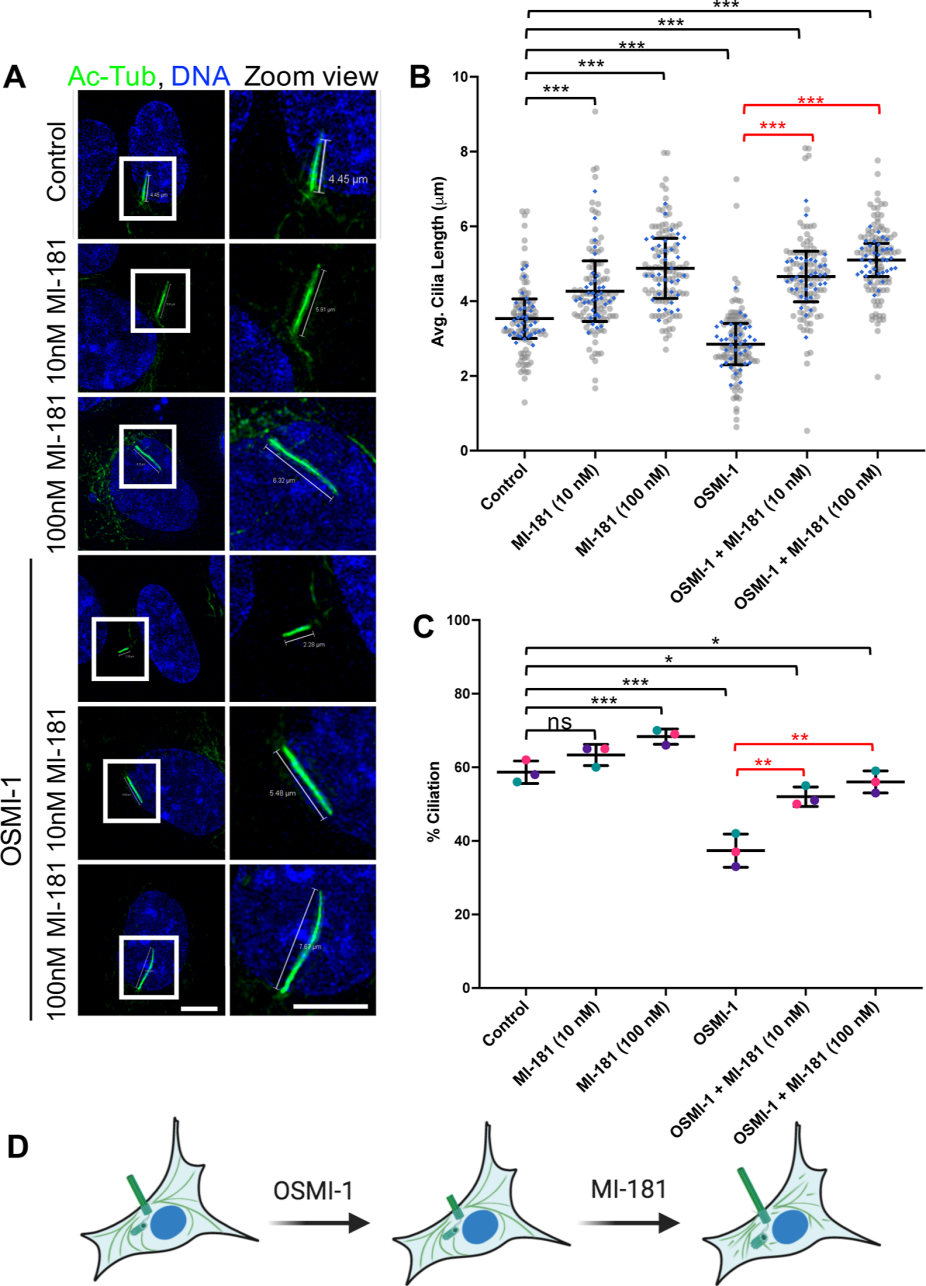
MI-181 restores ciliation and cilia length in cells with compromised shortened cilia. (A) IF microscopy of hTERT RPE-1 cells treated with DMSO, 10 nM MI-181, 100 nM MI-181, 25 μM OSMI-1, 25 μM OSMI-1 + 10 nM MI-181, or 25 μM OSMI-1 + 100 nM MI-181 for 24 h after serum withdrawal, fixed, and costained for cilia (acetylated tubulin, green) and DNA (Hoechst 33342, blue). Right side panels show zoom view of the areas in the white boxes in the left panels. Scale bars indicate 5 μm. (B) Graph shows summary of the average length of cilia (*y*-axis) for each treatment (*x*-axis). (C) Graph shows summary of the percentage of ciliated cells (*y*-axis) for each treatment (*x*-axis). (B–C) Data is represented as the average ± SD. Asterisks indicate statistical significance as *p < 0.05, **p < 0.01, and ***p < 0.001 compared to control. Not statistically significant is indicated by ns. See Quantification and Statistical Analyses section for details. (D) Schematic summary of the results.

Mutations in the IFT machinery have been detected in patients with ciliopathies that are attributed to a reduction in cilia formation, shortened cilia, and nonfunctional cilia.^2^ Therefore, we sought to determine if MI-181 could restore cilia formation and cilia length in cells with a compromised IFT system, which display shorter cilia or lack cilia. To do this, we utilized the small molecule Ciliobrevin D, which inhibits the function of the Dynein-2 complex, an important component of the IFT system, and inhibits retrograde transport within cilia, leading to a decrease in the percentage of ciliated cells and shorter defective cilia.^14^ hTERT RPE-1 cells were treated with either DMSO, 50 μM Ciliobrevin D, 10 nM MI-181, 100 nM MI-181, 50 μM Ciliobrevin D + 10 nM MI-181, or 50 μM Ciliobrevin D + 100 nM MI-181 for 24 h after serum withdrawal and cilia were imaged by IF microscopy. Ciliobrevin D treatment alone led to a marked decrease in the average length of cilia (DMSO = 3.40 ± 0.68 μm, Ciliobrevin D = 1.22 ± 0.46 μm, *p* < 0.001) and the percentage of ciliated cells (DMSO = 59.67 ± 1.53, Ciliobrevin D = 21.00 ± 3.61, *p* < 0.001) (Figure 3A–C). However, the cilia length defects and decrease in percent ciliation observed in Ciliobrevin D treated cells were rescued to near control levels when cells were cotreated with 10 nM MI-181 (average cilia length: Ciliobrevin D = 1.22 ± 0.46 μm, Ciliobrevin D + 10 nM MI-181 = 3.90 ± 0.42 μm, *p* < 0.001; percent ciliation: Ciliobrevin D = 21.00 ± 3.61, Ciliobrevin D + 10 nM MI-181 = 41.33 ± 2.31, *p* < 0.01) or 100 nM MI-181(average cilia length: Ciliobrevin D = 1.22 ± 0.46 μm, Ciliobrevin D + 100 nM MI-181 = 4.06 ± 0.54 μm, *p* < 0.001; percent ciliation: Ciliobrevin D = 21.00 ± 3.61, Ciliobrevin D + 100 nM MI-181 = 44.67 ± 3.51, *p* < 0.01) (Figure 3A–C). These results indicated that MI-181 rescued ciliogenesis and cilia length defects in cells with a compromised IFT system (Figure 3D). Similar results were obtained when cells were treated with 25 μM OSMI-1, an O-GlcNAc transferase (OGT) inhibitor that induces shorter cilia,^15,16^ and cotreatment with MI-181 restored ciliation and cilia length (Figure 4A–D).

### MI-181 Restores the Localization of Cilia Markers in Cells with Compromised Shortened Cilia

**Figure 5:**
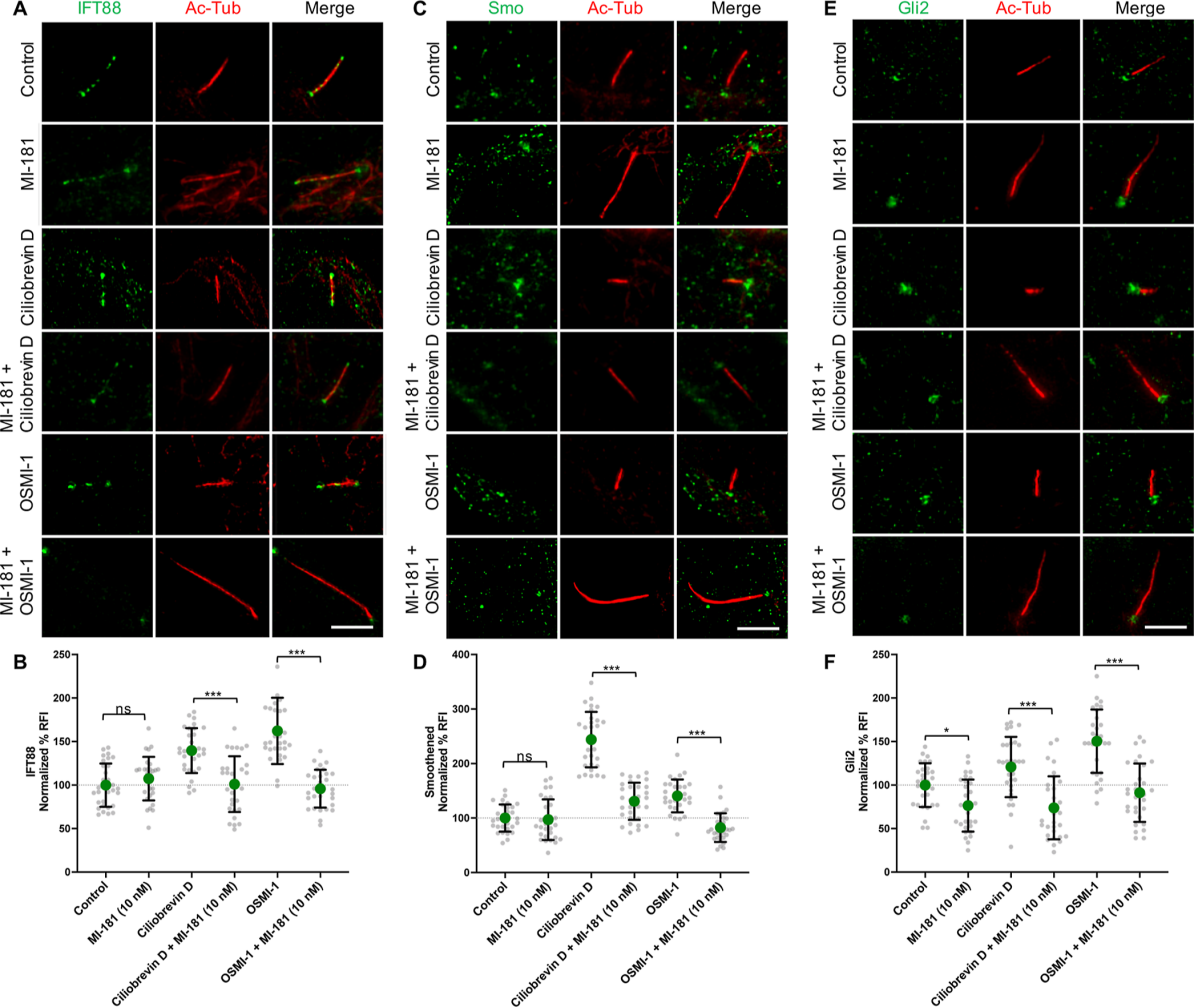
MI-181 restores the localization of cilia markers in cells with shortened cilia. (A, C, E) hTERT RPE-1 cells were treated with control DMSO, 10 nM MI-181, 50 μM Ciliobrevin D, 50 μM Ciliobrevin D + 10 nM MI-181, 25 μM OSMI-1, or 25 μM OSMI-1 + 10 nM MI-181 for 24 h after serum withdrawal, fixed, and costained for cilia (acetylated tubulin, red) and either IFT88 (A), Smo (C), or Gli2 (E). Scale bars indicate 5 μm. (B, D, F) Graphs show summary of the normalized percent RFI for IFT88 (B), smoothened (D) and Gli2 (F) at the base of the cilia (*y*-axis) for each treatment (*x*-axis). Data is represented as the average ± SD. Asterisks indicate statistical significance as *p < 0.05, **p < 0.01, and ***p < 0.001 compared to control. Not statistically significant is indicated by ns. See Quantification and Statistical Analyses section for details.

Due to the ability of MI-181 to induce ciliogenesis and restore cilia length in IFT compromised cells, we sought to determine if the distribution of the ciliary markers Intraflagellar Transport 88 (IFT88), Smoothened (Smo), and Gli family zinc finger 2 (Gli2) was similar in MI-181-treated versus nontreated cells. IFT88 is a core component of the IFT system and localizes to the base of cilia and throughout ciliary axonemes,^17^ Smo is a membrane G protein-coupled receptor important for the Hedgehog signaling pathway that localizes to the base of the cilia and subtly throughout the ciliary membrane,^18,19^ and Gli2 is a transcription factor important for Hedgehog target gene expression, which localizes to the base of cilia and subtly throughout the ciliary axonemes.^18,20^ hTERT RPE-1 cells were treated with DMSO, 10 nM MI-181, 50 μM Ciliobrevin D, or 50 μM Ciliobrevin D + 10 nM MI-181 for 24 h after serum withdrawal and cilia markers were imaged by IF microscopy. IFT88 localized to the base of cilia and throughout the ciliary axonemes in all conditions (Figure 5A). However, Ciliobrevin D treatment led to shorter cilia and an accumulation of IFT88 at the base of cilia, which was ameliorated when cells were cotreated with MI-181 (normalized percent relative fluorescence intensity (RFI) at the base of cilia: Ciliobrevin D = 140 ± 26, Ciliobrevin D + 10 nM MI-181 = 101 ± 32, *p* < 0.001) (Figure 5A, B). Interstingly, IFT88 localized throughout elongated cilia and at the tips in MI-181 treated cells, comparable to its localization in control cells (Figure 5A). Smo and Gli2 localized predominantly to the base of cilia in control and MI-181 treated cells (Figure 5C,E). However, Ciliobrevin D treated cells had shorter cilia and both Smo and Gli2 accumulated at the base of cilia, which was again ameliorated when cells were cotreated with MI-181 (Figure 5D,F). Together, these results indicated that MI-181 induced the restoration of cilia length in cells with a compromised IFT system and also allowed the distribution of ciliary markers to be restored. Similar shortened cilia and accumulation of ciliary markers at the base of cilia was observed in hTERT RPE-1 cells that had been treated with 25 μM OSMI-1 and cotreatment with MI-181 restored cilia length and the distribution of ciliary markers in the cilia (Figure 5A–F).

### MI-181’s Induced Increase in Cilia Length Persists Post Drug Washout

**Figure 6:**
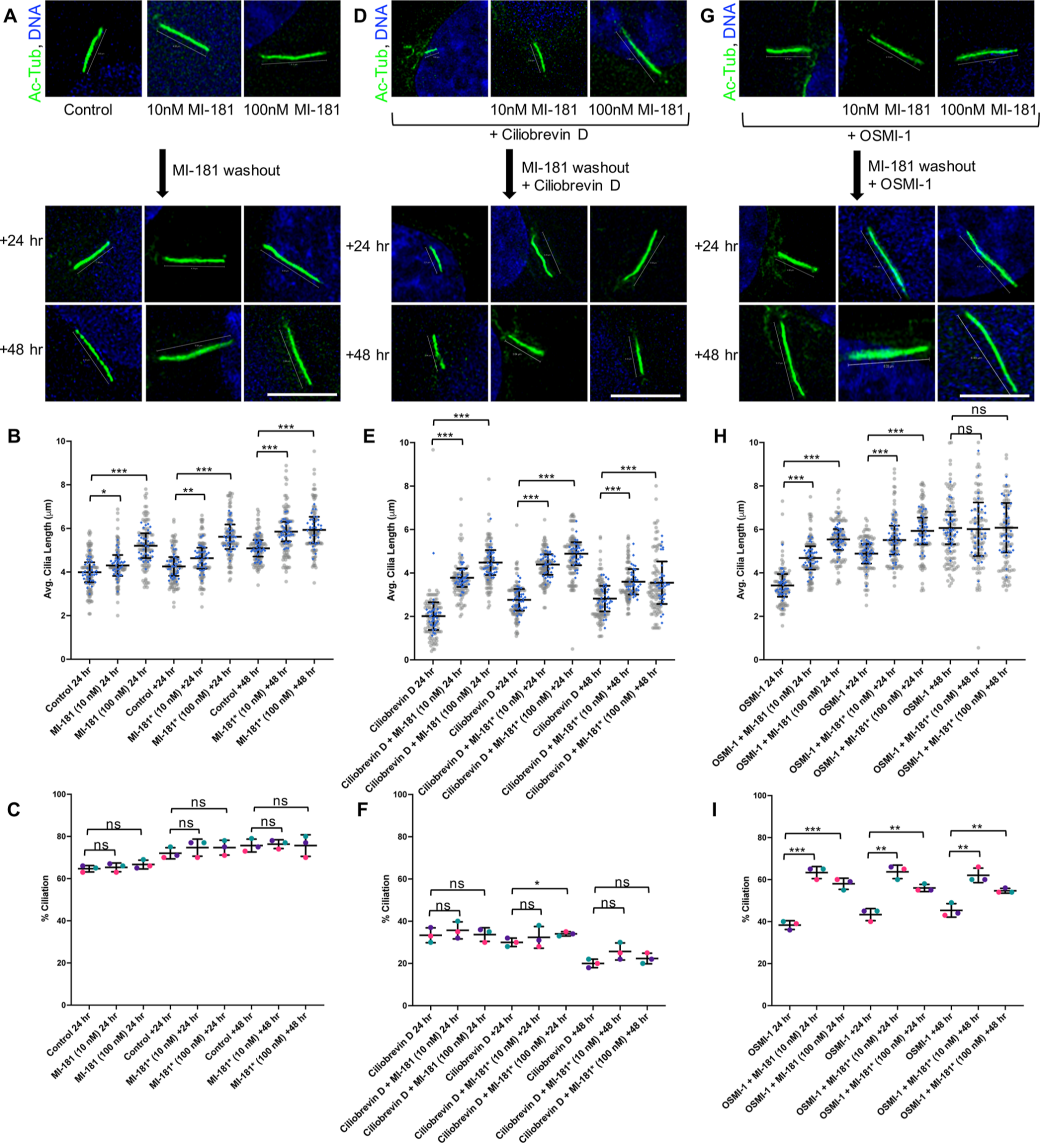
MI-181 induced increase in cilia length persists post MI-181 washout under conditions that compromise ciliation and cilia length. hTERT RPE-1 cells were treated with (A) DMSO, 10 nM MI-181, or 100 nM MI-181; (D) 50 μM Ciliobrevin D, 50 μM Ciliobrevin D + 10 nM MI-181, 50 μM Ciliobrevin D + 100 nM MI-181; (G) 25 μM OSMI-1, 25 μM OSMI-1 + 10 nM MI-181, or 25 μM OSMI-1 + 100 nM MI-181and allowed to form cilia for 24 h after serum withdrawal. MI-181 was then washed out (indicated by MI-181* in subsequent graphs) and cells were maintained in serum-free media for 24 or 48 h in the presence of either no drug (A), 50 μM Ciliobrevin D (D), or 25 μM OSMI-1 (G). Cells were then fixed, costained for cilia (acetylated tubulin, green) and DNA (Hoechst 33342, blue) and imaged by IF microscopy. Scale bars indicate 5 μm. (B, E, H) Graphs show summary of the average length of cilia (*y*-axis) for each treatment (*x*-axis). (C, F, I) Graphs show summary of the percentage of ciliated cells (*y*-axis) for each treatment (*x*-axis). (B, C, E, F, H, I) Data is represented as the average ± SD. Asterisks indicate statistical significance as *p < 0.05, **p < 0.01, and ***p < 0.001 compared to the indicated control. Not statistically significant is indicated by ns. See Quantification and Statistical Analyses section for details. MI-181* indicates that cells had been previously treated with MI-181 and MI-181 was washed out.

Next, we sought to determine if the increase in cilia length induced by MI-181 treatment could persist once MI-181 was washed out. To do this, we treated hTERT RPE-1 cells with DMSO, 10 nM MI-181, or 100 nM MI-181 for 24 h after serum withdrawal. Cells were then washed and maintained in drug and serum free media and cilia length was analyzed at 24 and 48 h post MI-181 washout. Cells were then fixed and stained for cilia and imaged by IF microscopy. At 24 and 48 h post MI-181 washout, the cilia remained longer in cells that had been previously treated with 10 or 100 nM MI-181 compared to the control DMSO treatment (average cilia length at 24 h post MI-181 washout (washout is denoted by *): DMSO = 4.25 ± 0.43, 10 nM MI-181* = 4.63 ± 0.48 *p* < 0.01, 100 nM MI-181* = 5.62 ± 0.56 *p* < 0.001; average cilia length at 48 h post MI-181 washout: DMSO = 5.08 ± 0.38, 10 nM MI-181* = 5.85 ± 0.45 *p* < 0.001, 100 nM MI-181* = 5.93 ± 0.60 *p* < 0.001) (Figure 6A,B). These results indicated that the effect that MI-181 had on cilia length persisted for at least 48 h post MI-181 washout.

Next, we asked if MI-181’s induced increase in cilia length could persist post MI-181 washout under conditions that lead to a decrease in ciliation and cilia length, i.e. Ciliobrevin D or OSMI-1 treatment. hTERT RPE-1 cells were treated with 50 μM Ciliobrevin D, 50 μM Ciliobrevin D + 10 nM MI-181, 50 μM Ciliobrevin D + 100 nM MI-181, 25 μM OSMI-1, 25 μM OSMI-1 + 10 nM MI-181, or 25 μM OSMI-1 + 100 nM MI-181 for 24 h after serum withdrawal. Cells were then washed to remove only MI-181, Ciliobrevin D and OSMI-1 were maintained as indicated. At 24 and 48 h post MI-181 washout, cells were fixed and stained for cilia and imaged by IF microscopy. For both the Ciliobrevin D and OSMI-1 treated sets, cells that had been previously treated with MI-181, and washed out, maintained longer cilia than cells that had not received MI-181 at both the 24 and 48 h time points post MI-181 washout (Figure 6D, E, G, H). For example, the average length of cilia at 24 h post MI-181 washout (* denotes washout) for the Ciliobrevin D treated set was: Ciliobrevin D = 1.97 ± 0.62 μm, Ciliobrevin D + 10 nM MI-181* = 3.69 ± 0.41 μm, *p* < 0.001, Ciliobrevin D + 100 nM MI-181* = 4.37 ± 0.56 μm, *p* < 0.001 (Figure 6D, E). These results were again consistent with the idea that the effect of MI-181 on cilia length persisted post MI-181 washout.

## Conclusions

Primary cilia are important microtubule-based organelles that coordinate extracellular environment sensing with cellular homeostasis and differentiation pathways.^1^ Dysregulation of ciliogenesis and/or ciliary length control can lead to an array of ciliopathies.^1^ Currently, there is a pressing need to define novel drugs that can be used to treat ciliopathies.

Both ciliation and cilia length control have been proposed as potential therapeutic targets for developing treatments for disorders characterized by the lack of cilia and defects in cilia length.^21^ Our results indicate that MI-181 is a viable path forward toward developing pharmacological treatments for ciliopathies. First, MI-181 is potent at inducing an increase in cilia length; concentrations as low as 10 nM significantly increase the length of cilia and the percentage of ciliated cells within a population of cells. Second, MI-181 is potent at restoring cilia length in cells with defective shortened cilia. Third, MI-181 does not appear to have a negative concentration-dependent response with regard to ciliogenesis and cilia length control, like other microtubule-targeting agents. Finally, MI-181’s effect on cilia length persists for some time post MI-181 washout. Together, our results show proof of principle that MI-181 could be used to induce ciliogenesis and restore cilia length. To further the translation of MI-181 as a therapeutic, future studies could focus on whether MI-181 can restore ciliogenesis, cilia length, and cilia function in human ciliopathy disease models. Additionally, our study focused on nonmotile cilia and it would be of interest to see if MI-181 also translates to improving cilia length and/or function of motile cilia, which could address respiratory illnesses like mucociliary diseases that are characterized by defective motile cilia.^22^

## Methods

### Cell Culture

hTERT RPE-1 cells (ATCC, verified by short-tandem repeat profiling) were cultured in DMEM/F12 1:1 (Cytiva) with 10% FBS (Gibco) in 5% CO_2_ at 37 °C. Cells were induced to ciliate by growing them to ∼90% confluency, then washed twice with phosphate-buffered saline (PBS), and further cultured in DMEM/F12 1:1 without FBS for 24–48 h. Please see Table S1 for a list of medias, cell lines, antibodies, chemicals, and software used in this study and their identifying information.

### Compound Treatments

Ciliated or nonciliated hTERT RPE-1 cells were treated with the indicated concentrations of MI-181 (Enamine), 100 nM Paclitaxel (Sigma), 166 nM Nocodazole (Sigma), 10 μM Colchicine (Selleckchem), 50 μM Ciliobrevin D (Selleckchem), and 25 μM OSMI-1 (Sigma), separately or in tandem, for 24 or 48 h before fixation.

### Immunofluorescence Microscopy

For IF microscopy, either ciliated or nonciliated hTERT RPE-1 cells (treated or untreated) were fixed with 4% paraformaldehyde, permeabilized with 0.2% Triton X-100/PBS, and blocked with IF buffer (PBS, 5% fish gelatin, 0.1% Triton X-100) before being incubated with 0.5 mg mL^–1^ Hoechst 33342 and the indicated primary antibodies in IF buffer at RT for 1 h. Cells were then washed with PBS three times, 5 min each, and incubated with secondary antibodies in IF buffer for 30 min. After a final wash, the coverslips were mounted with ProLong Gold Antifade mounting solution (Thermo Fisher Scientific) on glass slides. Images were captured with a Leica DMI6000 microscope (Leica Microsystems, 63*x*/1.40 NA oil objective, Leica Application Suite AF6000 software) or a Leica MICA microscope (Leica Microsystems, 63*x*/1.40 NA oil objective, Leica Application Suite X software) and exported as TIFF files. For IF microscopy experiments specifically using anti-Smoothened and anti-IFT88 antibodies, either ciliated or nonciliated hTERT RPE-1 cells (treated or untreated) were fixed with 100% methanol, followed by subsequent primary and secondary antibodies. For IF microscopy experiments specifically using anti-Gli2 antibody, either ciliated or nonciliated hTERT RPE-1 cells (treated or untreated) were fixed with 4% paraformaldehyde prepared in cytoskeletal buffer (CB, pH 6.9) (100 mM NaCl, 300 mM sucrose, 3 mM MgCl_2_, and 10 mM PIPES). Immediately before use, for a 50 mL volume of CB, 250 μL of Triton X-100 and 250 μL of 1 M EGTA were added.

### Quantification and Statistical Analyses

For cilia length measurements, three independent experiments were conducted for each condition, measuring cilia length in 35 cells per experiment (n = 105) using Leica AF6000/LAS-X software. For percent ciliation measurements, three independent experiments were performed per condition, observing 100 cells per experiment (*n* = 300) for ciliated versus nonciliated cells. Three replicates were averaged and analyzed using the General Linear Model (GLM). As a preliminary analysis, two-sample tests were used, and normal probability plots were checked before applying the GLM to ensure data normality. For RFI measurements of cilia markers at the base of cilia, 30 cells were analyzed with a 1 μm × 1 μm box centered at the base. Percent ciliation data were analyzed using an unpaired Student’s *t*-test, with statistical significance set at an adjusted *p*-value < 0.05 for multiple comparisons. Comparisons against the control group were made using 1-way or 2-way ANOVA within the GLM, followed by posthoc analyses if the ANOVA showed significant, differences. Dunnett’s procedure was used for comparisons to the control group (Figures 1, S1, 2, S2, S3), and Bonferroni corrections were applied for prespecified multiple comparisons (Figures 3–5). Statistical significance after multiple comparison adjustments is indicated by asterisks: *p < 0.05, **p < 0.01, ***p < 0.001. Data graphs were generated with GraphPad Prism 5 and are presented as mean ± SD. All analyses were performed using SAS 9.4. See Table S2 for all statistical analyses.

## Supporting Information Available

Supporting Information Figures S1, S2, and S3, and Tables S1 and S2 described in the main text (PDF)
(XLSX)
