# Supplementary material for: MI-181 Modulates Cilia Length and Restores Cilia Length in Cells with Defective Shortened Cilia
Source: ACS Chem Biol. 2024 Aug 6;19(8):1733–42. doi: 10.1021/acschembio.4c00186 (PMC11334112; doi:10.1021/acschembio.4c00186)
Supplement: Supplementary file 1 — cb4c00186_si_001.pdf [file cb4c00186_si_001.pdf]

Supporting Information

MI-181 Modulates Cilia Length and Restores Cilia Length in Cells with Defective Shortened Cilia

Ankur A. Gholkar[1], Thomas V. Gimeno[1], Jalie E. Edgemon[1], Myung Shin Sim[2,3], Jorge Z. Torres[1,4,5*]

[1]Department of Chemistry and Biochemistry, [2]Department of Medicine Statistics Core,

[3]Department of Medicine's Division of General Internal Medicine and Health Services Research,

[4]Jonsson Comprehensive Cancer Center, [5]Molecular Biology Institute, University of California,

Los Angeles, CA 90095.

***Correspondence to:**

Jorge Z. Torres

UCLA Department of Chemistry and Biochemistry

Los Angeles, CA 90095

Phone: 310-206-2092

torres@chem.ucla.edu

Table of Contents

Figure S1. Analysis of ciliation and cilia length by immunofluorescence (IF) microscopy in non-starved hTERT RPE-1 cells treated with DMSO, MI-181, colchicine, taxol, or nocodazole…..S3

Figure S2. Analysis of ciliation and cilia length by IF microscopy in hTERT RPE-1 cells treated with DMSO or increasing concentrations of MI-181 for 2 hours……………………………….S4

Figure S3. Analysis of ciliation and cilia length by IF microscopy in non-starved hTERT RPE-1 cells treated with DMSO or increasing concentrations of MI-181 for 24 hours………………..S5

Table S1. List of reagents and resources used in this study……………...……….………......S6

Table S2. Data statistical analysis…………………………………………………………….S7

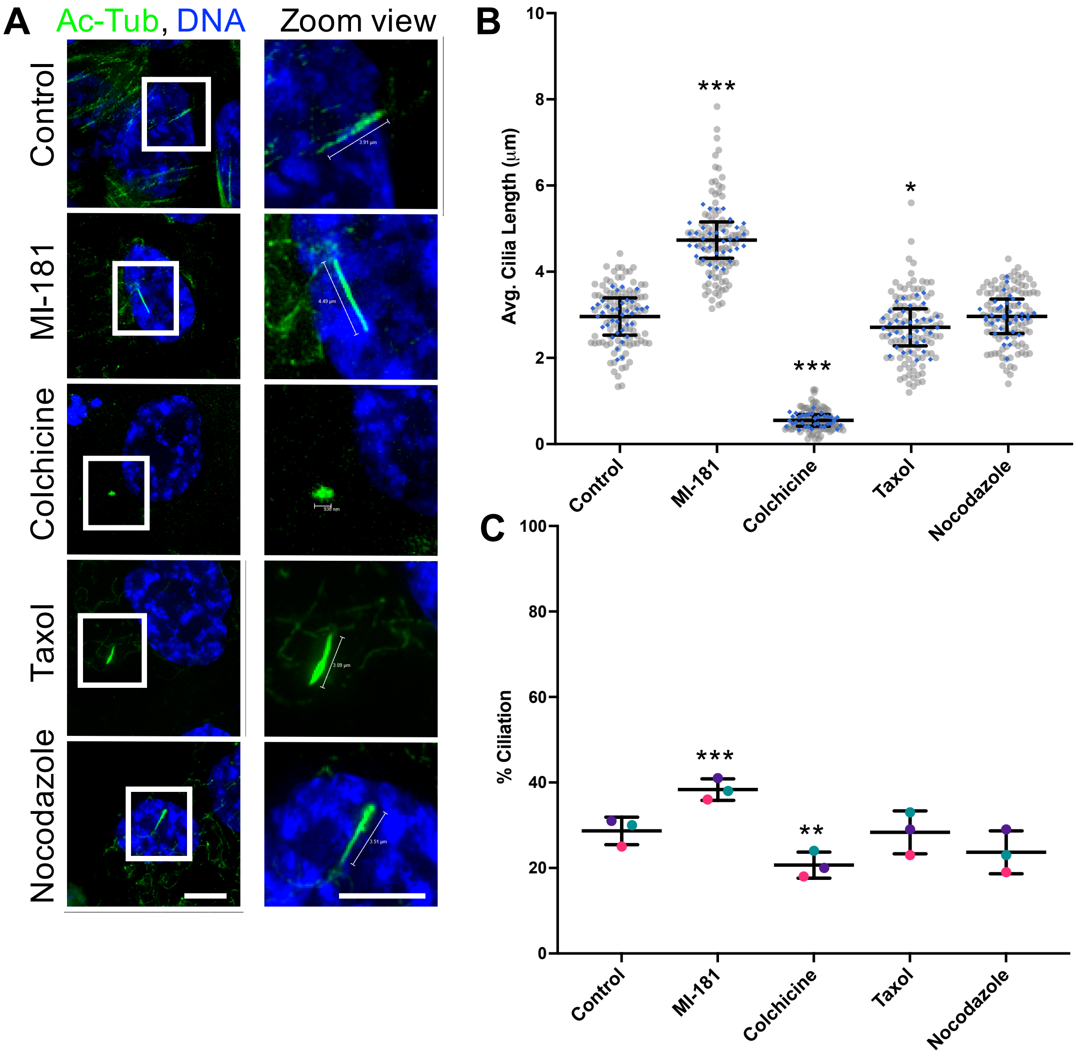

Figure S1. MI-181 modulates cilia length in non-starved cells. (A) Immunofluorescence (IF) microscopy of hTERT RPE-1 cells treated with control vehicle DMSO, 100 nM MI-181, 10 µM colchicine, 100 nM taxol, or 116 nM nocodazole for 24 hours, fixed, and co-stained for cilia (anti-acetylated tubulin, green) and DNA (Hoechst 33342, blue). Right side panels show zoom view of the areas in the white boxes in the left panels. Scale bars indicate 5 µm. (B) Graph shows summary of the average length of cilia (y-axis) for each treatment (x-axis). (C) Graph shows summary of the percentage of ciliated cells (y-axis) for each treatment (x-axis). (B-C) Data is represented as the average ±SD. Asterisks indicate statistical significance as * $p<0.05$, ** $p<0.01$, and *** $p<0.001$ compared to control.

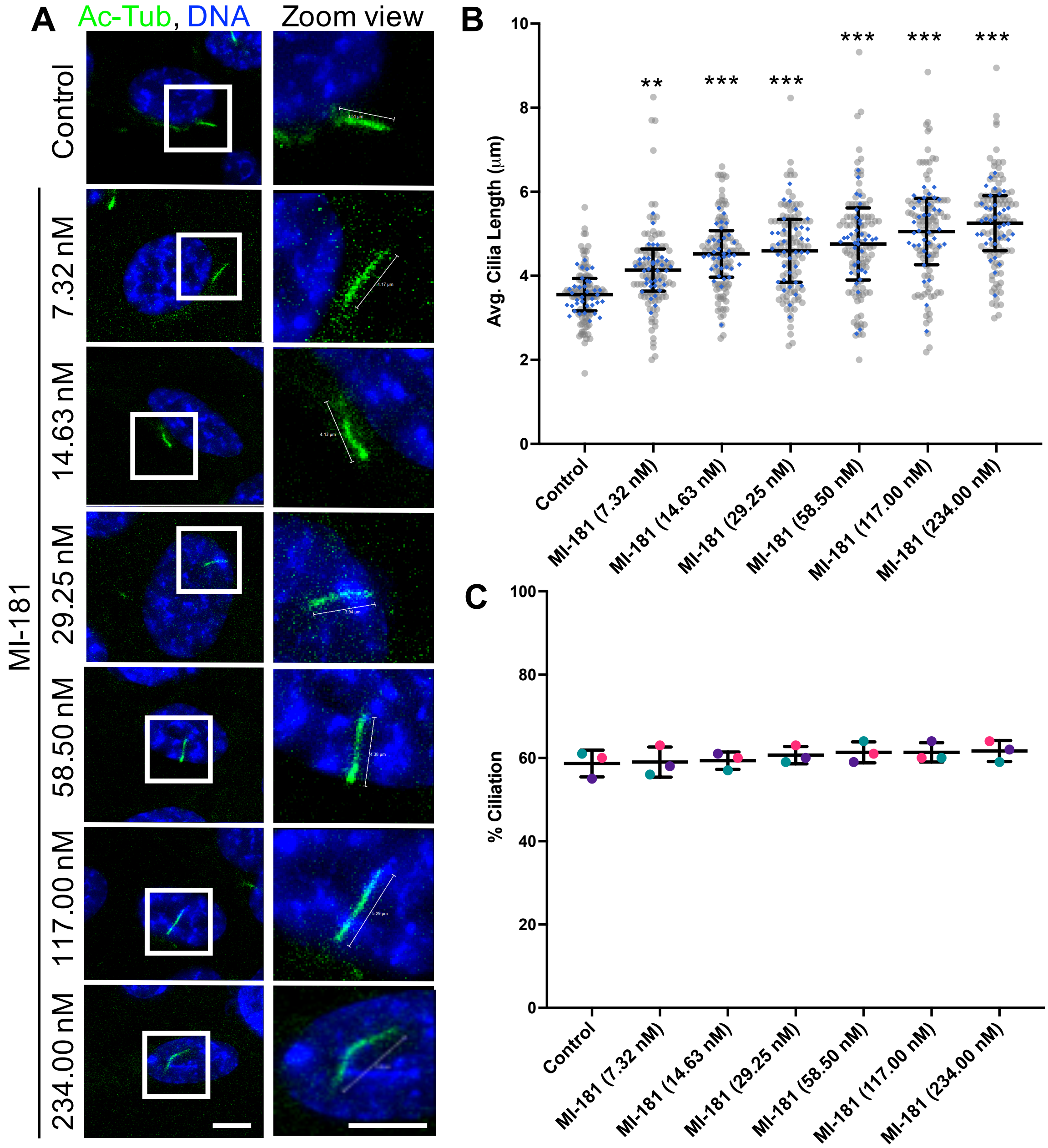

Figure S2. Two-hour treatment of MI-181 modulates cilia length in a concentration-dependent manner. (A) hTERT RPE-1 cells were induced to ciliate upon serum withdrawal for 24 hours. Ciliated cells were treated with control DMSO or increasing concentrations of MI-181 (7.32 nM to 234 nM) for 2 hours, fixed, and co-stained for cilia (acetylated tubulin, green) and DNA (Hoechst 33342, blue). Scale bars indicate 5 µm. (B) Graph shows summary of the average length of cilia (y-axis) for each treatment (x-axis). (C) Graph shows summary of the percentage of ciliated cells (y-axis) for each treatment (x-axis). (B-C) Data is represented as the average ±SD, asterisks indicate statistical significance as ** $p<0.01$ and *** $p<0.001$ compared to control.

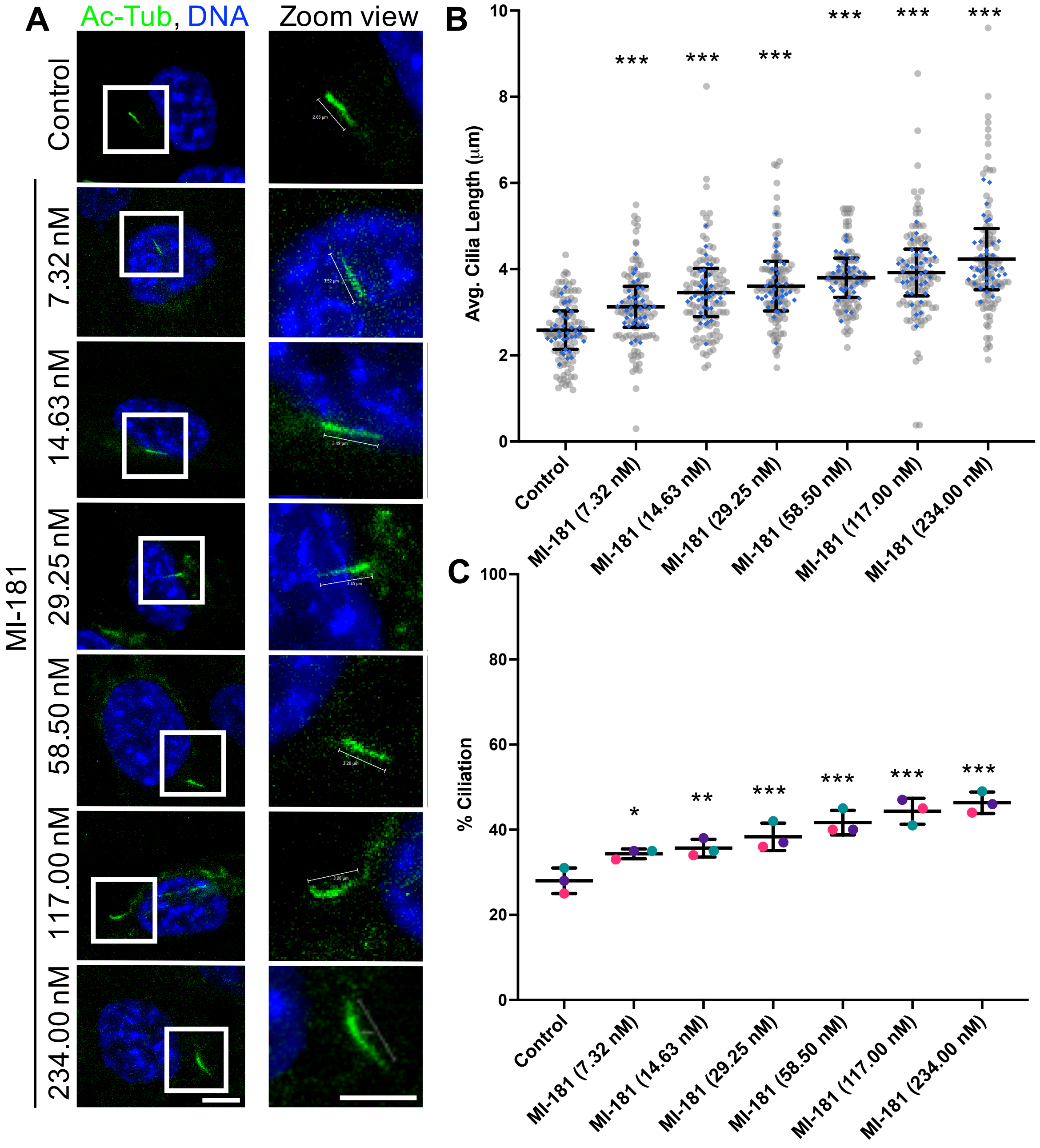

Figure S3. MI-181 modulates ciliation and cilia length in non-starved cells in a concentration-dependent manner. (A) IF microscopy of hTERT RPE-1 treated with control DMSO or increasing concentrations of MI-181 (7.32 nM to 234 nM) for 24 hours, fixed, and co-stained for cilia (acetylated tubulin, green) and DNA (Hoechst 33342, blue). Right side panels show zoom view of the areas in the white boxes in the left panels. Scale bars indicate 5 μm. (B) Graph shows summary of the average length of cilia (y-axis) for each treatment (x-axis). (C) Graph shows summary of the percentage of ciliated cells (y-axis) for each treatment (x-axis). (B-C) Data is represented as the average ±SD, asterisks indicate statistical significance as * $p<0.05$, ** $p<0.01$, and *** $p<0.001$ compared to control.

Table S1.

REAGENT or RESOURCE	SOURCE	IDENTIFIER
Antibodies		
Mouse monoclonal anti-acetylated-Tubulin (clone 6-11B-1)	Sigma	Cat# T6793<br>RRID: AB_477585
Rabbit polyclonal anti-Smoothened	Abcam	Cat# ab236465<br>RRID: AB 2935839
Rabbit polyclonal anti-IFT88	Proteintech	Cat# 13967-1-AP<br>RRID: AB_2121979
Rabbit polyclonal anti-Gli2	Proteintech	Cat# 18989-1-AP<br>RRID: AB_10596479
Donkey polyclonal anti-Rat IgG (H+L), Cy3 AffiniPure	Jackson ImmunoResearch Labs	Cat# 712-165-153<br>RRID: AB_2340667
Donkey polyclonal anti-Rat IgG (H+L), Cy5 AffiniPure	Jackson ImmunoResearch Labs	Cat# 712-175-150<br>RRID: AB_2340671
Donkey polyclonal anti-Mouse IgG (H+L), Fluorescein (FITC) AffiniPure	Jackson ImmunoResearch Labs	Cat# 715-095-151<br>RRID: AB_2335588
Donkey polyclonal anti-Mouse IgG (H+L), Cy3 AffiniPure	Jackson ImmunoResearch Labs	Cat# 715-165-151<br>RRID: AB_2315777
Donkey polyclonal anti-Rabbit IgG (H+L), Fluorescein (FITC) AffiniPure	Jackson ImmunoResearch Labs	Cat# 711-095-152<br>RRID: AB_2315776
Chemicals		
ProLong Gold Antifade Mountant	Thermo Fisher Scientific	Cat# P36934
Hoechst 33342	Thermo Fisher Scientific	Cat# H1399<br>CAS: 23491-52-3
DMSO	Corning	Cat# 25-950-CQC<br>CAS: 67-68-5
Paclitaxel	Sigma-Aldrich	Cat# T7191<br>CAS: 33069-62-4
Nocodazole	Sigma-Aldrich	Cat# 1404<br>CAS: 31430-18-9
Colchicine	Selleckchem	Cat# S2284<br>CAS: 64-86-8
Ciliobrevin D	Selleckchem	Cat# S9743<br>CAS: 1370554-01-0
OSMI-1	Sigma-Aldrich	Cat# SML1621<br>CAS: 1681056-61-0
MI-181	Enamine	Cat# Z46083298
Cell Lines and Media		
hTERT RPE-1 cell line	ATCC	Cat# CRL-4000<br>RRID: CVCL_4388
DMEM/F12	Cytiva	Cat# SH30023.FS
10% FBS	Gibco	Cat# 16000044
Software and Algorithms		
Leica Application Suite (LAS) AF6000	Leica Microsystems	RRID:SCR_016555
Leica Application Suite (LAS) X	Leica Microsystems	RRID:SCR_013673
GraphPad Prism 5	GraphPad	RRID: SCR_002798
ImageJ	NIH ImageJ	https://imagej.nih.gov/ij/index.html
BioRender	BioRender	RRID: SCR_018361

Table S2. Data statistical analysis. Table S2 includes data summary and statistical significance analysis of data used to generate all graphs for all figures.
